# Level of Adherence to Prophylactic Osteoporosis Medication amongst Patients with Polymyalgia Rheumatica and Giant Cell Arteritis: A Cross-Sectional Study

**DOI:** 10.1155/2015/783709

**Published:** 2015-09-29

**Authors:** A. Emamifar, Rannveig Gildberg-Mortensen, S. Andreas Just, N. Lomborg, R. Asmussen Andreasen, I. M. Jensen Hansen

**Affiliations:** Department of Rheumatology, University Hospital of Odense, Svendborg Hospital, Valdemarsgade 51, 5700 Svendborg, Denmark

## Abstract

*Objective*. To estimate level of adherence to oral calcium and vitamin D supplementation as well as bisphosphonate amongst patients with PMR and GCA treated with glucocorticoids. *Method*. A total of 138 patients with the diagnosis of PMR and/or GCA registered in our department in December 2013. In this cross-sectional study we interviewed all the patients to measure level of adherence to calcium and vitamin D, as well as bisphosphonates. *Results*. Out of the 118 included patients, 88.9% of them were adherent to their prescription. Only 2 patients (1.7%) did not take calcium and vitamin D at all and 10 patients (8.5%) took their medication infrequently, 9 and 1 out of 10 patients took the medication 50–100% of the time and less than 50% of the prescribed dose, respectively. Sixty-one patients received additional treatment with bisphosphonate and 96.6% were adherent to this therapy. The remaining 3.4% of the patients did not take the medication at all. Forgetfulness, adverse side effects, and lack of understanding of treatment benefits were the most significant causes for nonadherence to calcium and vitamin D. *Conclusions*. Contrary to what we expected this study found that adherence to osteoporosis preventive medication in patients with PMR and GCA was high.

## 1. Introduction

Polymyalgia rheumatica (PMR) and Giant Cell Arteritis (GCA) are common inflammatory conditions that almost exclusively affect patients older than 50 years. These conditions often occur together. 18–26% of patients with PMR have GCA while 27–53% of patients with GCA have also PMR at the same time. PMR primarily presents with stiffness and pain in the proximal muscle including neck, shoulders, buttocks, and thighs that causes severe disability without suitable treatment. GCA is the most common vasculitis in older persons and involves medium to large artery. It can result in blindness if not diagnosed and treated immediately. Older age, female sex, and northern European descent are the most important risk factors for both conditions. Diagnosis is based on clinical and laboratory evaluations. Temporal artery biopsy should be performed in GCA cases [1, 2], patients with cranial symptoms, and when either diagnosis is suspected as proposed by the National Danish Guidelines [3]. Glucocorticoids (GC) are the mainstay of treatment in patients with PMR and GCA [4].

One of the important complications amongst PMR and GCA patients who received GC in the long term (>3 months) is glucocorticoid-induced osteoporosis (GIO) that leads to an increased risk of fracture. This is due to impairment of calcium absorption in the intestine and reabsorption in the tubular system of kidneys. Inflammation, reduced mobility, and older age which characterize this group of patients further contribute to an increased risk [2, 4, 5]. According to several studies bisphosphonates, vitamin D, and calcium are effective for preventing GIO [2, 4, 6–10]. The American College of Rheumatology suggests use of 1200–1500 mg/day calcium plus 800–1000 U/day vitamin D to prevent GIO. Furthermore lifestyle modification and regular weight bearing exercise can improve outcomes [5].

To prevent loss of bone mass, all of our patients with PMR and GCA were prescribed calcium and vitamin D supplements and depending on the bone density, evaluated with a DXA-scan (dual energy X-ray absorptiometry), received additional treatment with bisphosphonate (if *T*-score < −1). Previous studies showed that the level of adherence to osteoporosis preventive medications is poor, leading to increased risk of fracture and hospitalization [11–16]. Several factors may contribute to nonadherence including concern of potential side effects, inconvenience, cost of medication, or lack of understanding of the benefits of therapy [12, 16, 17]. The aim of the current study is to estimate adherence to oral calcium and vitamin D supplementation as well as bisphosphonate amongst patients with PMR and GCA receiving long-term treatment with GC and identify factors associated with nonadherence. In addition we evaluated patients' adherence when responsible physician referred the patients to a DXA-scan.

## 2. Method

We used patient interview to measure adherence to prescribed medication in a cross-sectional study. All patients with the diagnosis of PMR and/or GCA diagnosed according to the Danish national guideline, registered in the department of rheumatology, Svendborg hospital, in December 2013 were identified and contacted by phone and were asked if they wanted to participate in the study. They were requested to answer a set of previously defined questions (see Adherence Interview Form) about medication and adherence. All telephone interviews were performed by registered physicians or nurses who the patients knew. For each patient demographic data including age, sex, and smoking status were collected. Information on GC treatment, calcium, and vitamin D supplements and bisphosphonate was obtained using patients' records. Patients' records were also evaluated individually for previous laboratory tests, imaging studies including DXA-scan, positron emission tomography, and biopsy of temporal artery to ensure accurate diagnosis and if relevant diagnostic tests were performed. A patient was considered to be adherent if medication was taken as prescribed in the records. Finally the patients were asked about the reasons for nonadherence if they had discontinued the medications. The study was approved by the Danish Data Protection Agency (file number 2008-58-0035, 14/4017). All analyses were performed using SPSS version 22.0.

*Adherence Interview Form*

(1) Patient code number:
(2) Age:
(3) Diagnose:
   PMR
   GCA
(4) Smoking status
   Yes
   No
(5) Do you take prednisolone tablet daily:
   Yes
   No
(6) Prednisolone Dosage:
(7) Are you prescribed calcium and vitamin D supplements with start of prednisolone:
   Yes
   No
(8) Do you always remember to take your calcium, vitamin D supplements?
   100%
   50–100%
   <50%
   Never
(9) If yes, which type of calcium and vitamin D supplements do you take?
(10) Number of calcium and vitamin D tablets per day:
(11) If no, why don't you take your medication?
   Fear of side effects
   I don't take much medication
   I don't have more pills
   Side effects (nausea, constipation)
   I don't think it is necessary
   A doctor or nurse have told me not to eat it
   I can not afford it
   I don't know
   Others
(12) Do you eat dairy products or fish daily:
   Yes
   No

(13) Do you take vitamin supplements
   Yes
   No
(14) Have you previously done DXA-scan:
   Yes
   No
(15) If yes, date/year of previous DXA-scan:
(16) Do you take prophylactic osteoporosis medication for example Fosamax, Alendronate, Bonviva, Aclasta:
   Yes
   No
(17) If yes, name of your prophylactic osteoporosis medication:
(18) Do you take always your preventive medication:
   100%
   50–100%
   <50%
   Never
(19) Why do not you take your preventive medication:
   Fear of side effects
   I don't take much medication
   I don't have more pills
   Side effects
   I can not afford it
   I don't think it is necessary
   I don't know
   Others

## 3. Results

**Figure 1:**
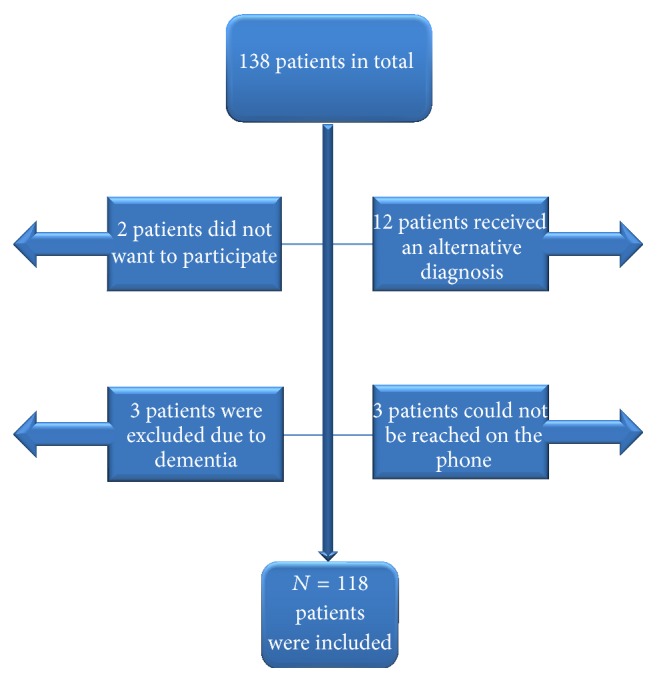
Patients included in the study.

We identified 138 patients with PMR and/or GCA. Twelve patients subsequently received an alternative diagnosis, in most cases Rheumatoid Arthritis, three patients could not be reached on the phone, two did not want to participate in the study, and three were not contacted, because it was clear from the records that they were demented. In total 118 patients were included (Figure 1).

**Figure 2:**
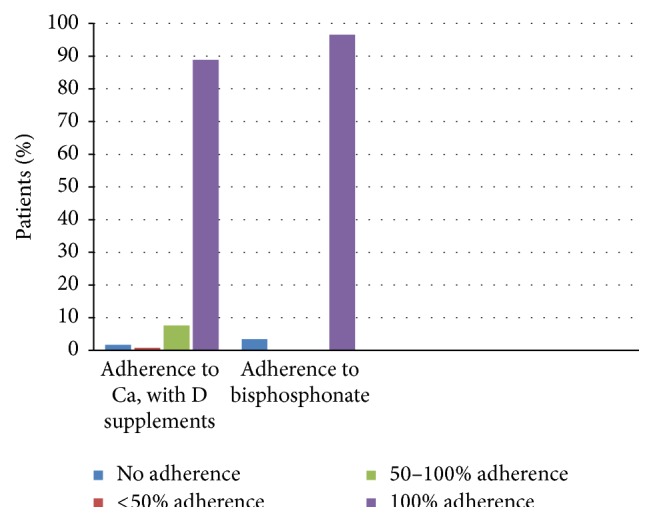
Level of adherence to Ca, vitamin D supplements, and bisphosphonates amongst PMR/GCA patients.

The mean age of patients was 73 ± 8 years. Sixty percent were female and 15% of patients were smoker. Out of 118 patients 85 patients were diagnosed with PMR and 33 patients were diagnosed with GCA. The mean of prednisolone dosage was 10.5 ± 9.4 mg with a range between 2.5 and 50 mg. 117 patients (99.2%) had prescribed calcium and vitamin D and 88.9% of them were adherent to their prescription. Only 2 patients (1.7%) did not take calcium and vitamin D at all, and 10 patients (8.5%) took their medication infrequently, 9 and 1 out of 10 patients took the medication 50–100% of the time and less than 50% of the prescribed dose, respectively (Figure 2).

**Figure 3:**
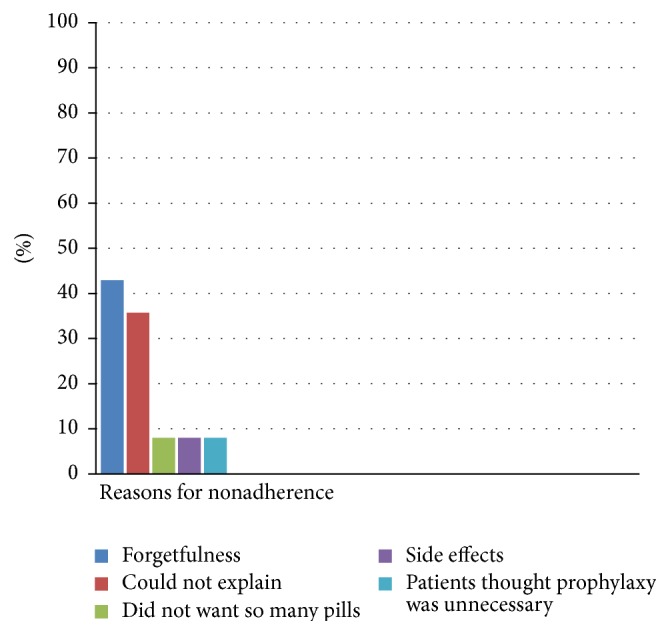
The most important causes of nonadherence amongst PMR/GCA patients.

The reasons for incomplete adherence were forgetfulness in 42.9% of patients, 35.7% of patients could not explain the reason, one patient did not want to eat so many pills, one patient claimed of side effects, and one patient thought prophylactic calcium and vitamin D supplement was unnecessary (Figure 3).

From the 118 patients 113 (95.7%) were referred to a DXA-scan, of which 103 had been scanned and 10 were awaiting scan results. 5 patients were not referred. All patients who were referred for DXA-scan were involved in the study. In 61 patients bisphosphonate was additionally prescribed and 96.6% were adherent to this therapy. The remaining 3.4% of the patients did not take the medication at all (Figure 2). Two patients discontinued the medication after receiving advice from their private physician. Nonadherence to bisphosphonate was in all cases due to gastrointestinal side effects.

## 4. Discussion

Glucocorticoid-induced osteoporosis is an important side effect of treatment with GC amongst patients with PMR and GCA. The frequency of osteoporosis ranges between 14.9 and 85% in patients. Risk of fracture in patients who received long-term GC is about 33–50% which depends on daily and cumulative dose [18]. Low body mass index, smoking, intake of more than two standard alcoholic drinks per day, and history of hip fracture in parents are related with higher risk for GIO [5]. As a result treatment with calcium and vitamin D should be started to prevent bone loss [4]. Bisphosphonates are generally indicated in patients receiving long-term GCs [5].

As in any medical treatment, adherence to prescribed medication is an important factor to achieve best outcome. Results of recent studies showed that adherence to osteoporosis preventive medications is relatively poor [11–13]. However, there has been some controversy regarding adherence to osteoporosis preventive medications. A cross-sectional study by Castelo-Branco et al. [11] in Spain showed that out of 7888 patients aged 45 or over, who received calcium and vitamin D supplementation, only 31.2% were adherent. Fatigue due to long-term therapy was the main reason for nonadherence amongst the patients. Another study by Rossini et al. [15] carried out on 9851 postmenopausal women revealed that adherence to treatment with osteoporosis preventive medications was poor. Additionally, the authors concluded that the most important reasons of nonadherence are fear of side effects and lack of motivation. On the contrary, recent study by Tafaro et al. [19] showed high adherence to osteoporosis preventive medications if therapies less than 30 days were excluded. Out of 6930 patients who were included in the study 43.8% were adherent to the treatment. The most frequent reasons of nonadherence were included side effects and misinformation given by the physician.

Contrary to what we expected this study found that adherence to preventative osteoporosis medications in patients with PMR and GCA was high and the most frequent causes for nonadherence were forgetfulness and side effects. One of the reasons of our patients' high adherence to the osteoporosis preventive medications is patients' awareness of GCs side effects and osteoporosis. This is due to the fact that all physicians are responsible to inform patients about the side effects of GC treatment. In the same way responsible physicians and nurses are asked to update the patient medical status at each visit and specifically ask whether the patients prescribed osteoporosis preventive medication while they receive GC treatment. When patients pick up their prescribed GC from the drugstore they will also be reminded that they should take calcium and vitamin D. In addition the important patient association, the Osteoporosis Associations in Denmark with 24 local subdivisions, gives useful information about osteoporosis to the patients who are at risk of osteoporosis as well as entire population. This is in line with previous findings [14, 16, 18], suggesting that the deficit in patient awareness is related with low adherence and physicians support is an important factor to achieve better adherence. On the other hand, most of our patients (96%) are referred to carry out DXA-scan which stressed the importance of treatment. Another important factor for the high adherence in our patient group could be that PMR/GCA can be very painful conditions, where the prescribed prednisolone treatment causes rapid freedom from symptoms. This can underpin confidence in the physician's treatment and why adherence to the additional prophylactic treatment might be higher.

The limitation of our study was that there was no way to control patients' answers as well as medications intake. Moreover the accuracy of results was dependent on patients' memory to report their adherence level retrospectively. We cannot rule out information bias, as there will probably be a tendency for the patients to provide what they believe to be socially accepted answers rather than the truth, especially with regard to behavioral aspects and health conditions associated with taboos [20]. In our situation a taboo might be the fact that the patient had not followed the doctor's advice. Since the patients were asked the name of the formulations and since the patients in a friendly tone were told that lack of adherence is a common and understandable state, we believe that we have predominantly received correct answers from our patients. We think that our concept of giving thorough information concerning the side effects of GC to the patients at each visit in the clinic followed by a prescription of DXA-scan is advisable for all departments to increase patients' adherence. We also think that our patient population with well characterized and initially painful diseases were more motivated for prophylaxis than otherwise healthy postmenopausal osteoporotic patients, where the adherence is described to be low [11–13]. A possible way for evaluation whether the patients pick up the medicine at the pharmacies might be to check this on the electronic journals medication module, which is possible, but it requires an additional permission from the Danish Data Protection Agency as well as from the patient. There is probably little doubt that this would astonish patients much and give them a feeling that we do not trust them, which would make cooperation with the patients difficult in the future. More studies in this field are needed to confirm our results, but we see already a problem in a prospective design, because this cannot be done without patients knowing it will be checked, if they collect the medication, thereby inducing a relative risk for another information bias.
